# Genetically Diverse Filoviruses in *Rousettus* and *Eonycteris* spp. Bats, China, 2009 and 2015

**DOI:** 10.3201/eid2303.161119

**Published:** 2017-03

**Authors:** Xing-Lou Yang, Yun-Zhi Zhang, Ren-Di Jiang, Hua Guo, Wei Zhang, Bei Li, Ning Wang, Li Wang, Cecilia Waruhiu, Ji-Hua Zhou, Shi-Yue Li, Peter Daszak, Lin-Fa Wang, Zheng-Li Shi

**Affiliations:** Chinese Academy of Sciences, Wuhan, China (X.-L. Yang, R.-D. Jiang, H. Guo, W. Zhang, B. Li, N. Wang, L. Wang, C. Waruhiu, Z.-L. Shi);; Dali University, Dali, China (Y.-Z. Zhang);; University of Chinese Academy of Sciences, Beijing, China (R.-D Jiang, H. Guo, N. Wang);; Yunnan Institute of Endemic Diseases Control and Prevention, Dali (Y.-Z. Zhang, J.-H. Zhou);; Wuhan University, Wuhan (S.-Y. Li);; EcoHealth Alliance, New York, New York, USA (P. Daszak);; Duke–NUS Graduate Medical School, Singapore (L.-F. Wang)

**Keywords:** filoviruses, Filoviridae, Rousettus spp., Eonycteris spp., viruses, natural reservoirs, fruit bats, genetic diversity, China, Yunnan Province, reservoir, zoonoses, lung tropism

## Abstract

Genetically divergent filoviruses detected in *Rousettus* and *Eonycteris* spp. bats in China exhibited 61%–99% nt identity with reported filoviruses, based on partial replicase sequences, and they demonstrated lung tropism. Co-infection with 4 different filoviruses was found in 1 bat. These results demonstrate that fruit bats are key reservoirs of filoviruses.

Filoviruses (family *Filoviridae*) are nonsegmented, negative-strand RNA viruses belonging to 3 genera: *Marburgvirus*, *Ebolavirus*, and *Cuevavirus*. *Marburgvirus* comprises 1 species, *Marburg marburgvirus,* which includes Marburg virus (MARV) and Ravn virus. *Ebolavirus* comprises 5 species, *Zaire ebolavirus* (ZEBOV), *Sudan ebolavirus*, *Bundibugyo ebolavirus*, *Taï Forest ebolavirus*, and *Reston virus* (RESTV). *Cuevavirus* comprises 1 species, *Lloviu cuevavirus* (*1*). Filovirus-associated diseases, especially those caused by ZEBOV and MARV, are recognized as a major threat to public health, causing high rates of death among humans and nonhuman primates.

Bats have been implicated as natural reservoirs for filoviruses (*2**,**3*) on the basis of serologic evidence from 19 bat species in 8 countries across Asia, Africa, and Europe *(**2**,**4**–**9*). In addition, filovirus RNA has been detected in 8 bat species from 7 countries in the same regions (*2**–**4**,**10**–**13*). Outbreaks of Marburg hemorrhagic fever among miners in Uganda in 2007 were traced to bat MARV (*11*). In addition, we previously discovered filovirus antibodies in several bat species in China (*14*). This finding was further confirmed by He et al., who detected filovirus RNA in brown fruit bats (*Rousettus leschenaultii*) in China (*10*). Considering the diversity of bat species in the world, long-term surveillance of bat filoviruses is essential for better understanding of distribution, diversity, and ecology of these viruses. We conducted a study to determine the diversity of filoviruses among bats in Yunnan Province, China.

## The Study

**Figure 1:**
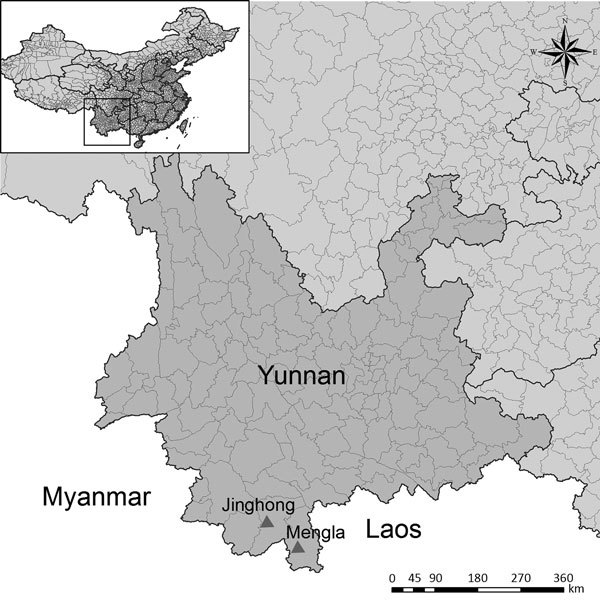
Bat collection sites for a study on genetically diverse filoviruses in *Rousettus* and *Eonycteris* spp. bats in China. Triangles indicate Jinghong City and Mengla County, Yunnan Province, where 150 apparently healthy adult bats were collected from 2 caves in November 2009 (Jinghong City) and December 2015 (Mengla County). Inset map shows the location of Yunnan Province in China.

We captured 150 apparently healthy adult bats from 2 caves in Yunnan Province, China: 1 in Jinghong City in November 2009, and 1 in Mengla County in December 2015 (Table 1; Figure 1). The bat species we collected were *Hipposideros armiger*, *Aselliscus stoliczkanus*, *Myotis ricketti*, *Rhinolophus Monoceros*, *Miniopterus fuscus*, *Ia io*, *Eonycteris spelaea*, and *Rousettus* sp. We humanely killed all bats and collected their hearts, intestines, lungs, spleens, kidneys, livers, brains, and blood for testing. We used 2 methods to analyze bat lung tissues for presence of filovirus RNA: first, we used nested PCR with the primers FV F1/R1 and FV F2/R2 (*10*), and next, we used quantitative PCR (qPCR) with 3 groups of qPCR with primers and probes designed from viral sequences obtained in this study (Technical Appendix Table).

**Table 1:** Filovirus infection detected in bat samples by PCR, ELISA, and Western blot, Yunnan Province, China, 2009 and 2015*

Bat species, by year and month of collection/location	No. positive/no. tested (%)						
	RT-PCR	Quantitative PCR	ELISA†			Western blot†	
			ZEBOV	RESTV		ZEBOV	RESTV
2009 Nov/Jinghong City							
* Hipposideros armiger*	0/15	0/15	0/15	0/15		0/15	0/15
* Rhinolophus monoceros*	0/4	0/4	0/4	0/4		0/4	0/4
* Ia io*	0/3	0/3	0/3	0/3		0/3	0/3
* Miniopterus fuscus*	0/1	0/1	0/1	0/1		0/1	0/1
* Myotis ricketti*	0/27	0/27	1/27 (3.7)	1/27 (3.7)		1/27 (3.7)	1/27 (3.7)
* Eonycteris spelaea *and *Rousettus* sp.	10/43 (23.3)	10/43 (23.3)	5/43 (11.6)	6/43 (13.9)		2/43 (4.6)	2/43 (4.6)
2015 Dec/Mengla County							
* Aselliscus stoliczkanus*	0/15	0/15	0/15	0/15		0/15	0/15
* E. spelaea *and *Rousettus* sp.	5/42 (11.9)	10/42 (23.8)	14/25 (56)	7/25 (28)		11/25 (44)	4/25 (16)

*EBOV, *Zaire ebolavirus*; RESTV, *Reston virus*. †ELISA and Western blot results for samples collected in 2009 were from a previous study (*14*).

**Figure 2:**
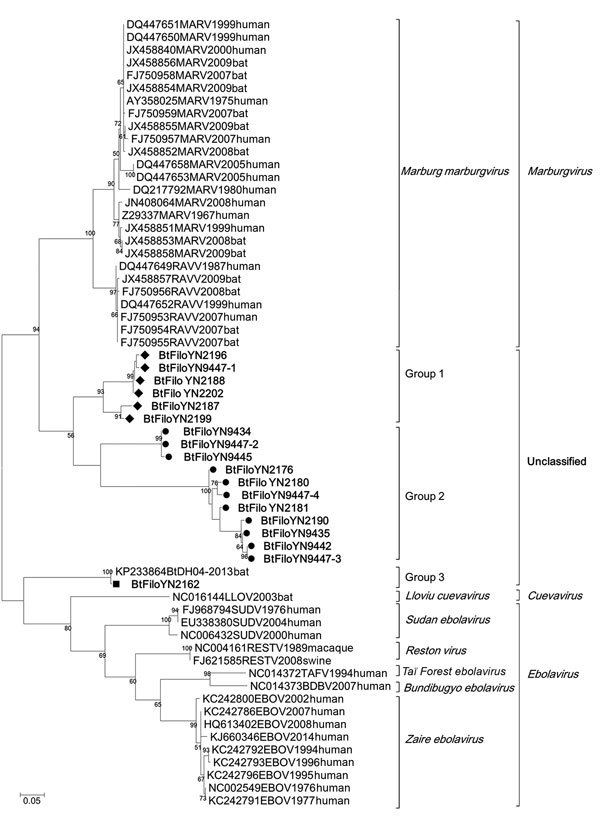
Phylogenetic analysis of filovirus isolates collected in study of genetically diverse filoviruses in *Rousettus* and *Eonycteris* spp. bats in China, compared with reference isolates. Analysis was based on a 310-bp segment of the filovirus L gene. Bootstrap values lower than 50 are not shown. The maximum-likelihood tree was constructed based on the 310-bp segment with 1,000 bootstrap replicates. The sequences obtained in this study are marked with a triangle (group 1), black dot (group 2), or rectangle [group 3). Sequences from GenBank are listed by their accession numbers, followed by the virus name, collection year, and host. Scale bar indicates nucleotide substitutions per site.

Using degenerate nested PCR, we detected filovirus RNA in 15 fruit bat specimens (*E. spelaea* and *Rousettus* sp.); the specimens comprised 10 (23.3%) of 43 *E. spelaea* and *Rousettus* sp. collected in 2009 and 5 (11.9%) of 42 collected in 2015. Using qPCR, we detected filovirus RNA in 20 specimens from *E. spelaea* (n = 4) and *Rousettus* sp. (n = 16) bats: 10 (23.3%) of the bats were collected in 2009 and 10 (23.8%) in 2015. No filovirus was detected in other bat species studied (Table 1). The 310-bp L gene sequences (GenBank accession nos. KX371873–KX371890) exhibited 65%–99% nt identity among themselves and 61%–99% nt identity with known filoviruses. Phylogenetic analysis showed that the sequences from the bats formed 3 independent groups, groups 1–3. Groups 1 and 2 comprised 6 and 11 sequences, respectively, all of which were obtained in this study (Figure 2). Group 3 comprised 2 sequences, 1 from this study and 1 previously published (*10*). Pairwise distance analysis indicated that sequences in group 1 share the highest nucleotide identity (75%–78%) with MARV and those in group 2 share the highest identity (69%–74%) with Ravn virus. The 2 sequences in group 3 are highly similar and share 66%–70% nt identity with other filovirus species. Of note, 1 bat specimen (no. 9447) was co-infected with 4 different filovirus strains (BtFiloYN9447–1 to 9447–4) with high divergence (Figure 2; Technical Appendix Figure 1). To further determine the phylogenetic relationship of these viruses with known filoviruses, we amplified more L gene sequence (1,475 bp) for strains BtFiloYN2162 and BtFiloYN9447–1. Similar to the 310-bp sequences, the 1,475-bp sequence of the BtfiloYN2162 shared 99% identity with BtDH04 at the nucleotide level, the 1,475-bp sequence of BtFiloYN9447–1 shared 62%–71% with known filoviruses.

To determine the tissue tropism of these viruses, we performed qPCR with primers and probes designed for each of the 3 different groups (online Technical Appendix Table). Results showed that filoviruses were mainly located in the lung and that genome copy numbers ranged from 29 to 523,582/mg of tissue (Table 2). Only 2 bat blood samples (nos. 2202 and 9447) were positive for filovirus RNA; 5 samples (nos. 2202, 2188, 9434, 9442, and 9447) contained filoviruses with more widespread tissue tropism. We were unable to isolate virus from PCR-positive samples by using Vero-E6 cells.

**Table 2:** Virus tropism and quantification in different tissues of *Eonycteris spelaea* and *Rousettus* sp. bats, China, 2009 and 2015

Sample no.	Species	Sex	Primer group*	Positive organs (viral genome copies/mg tissue or viral genome copies/μL blood)
2162	*E. spelaea*	M	3	Lung (119)
2176	*Rousettus* sp.	M	2	Lung (2,103)
2180	*Rousettus* sp.	F	2	Lung (42)
2181	*Rousettus* sp.	M	2	Lung (202)
2187	*Rousettus* sp.	F	1	Lung (46)
2188	*Rousettus* sp.	F	1	Lung (43), liver (400), kidney (42)
2190	*Rousettus* sp.	M	2	Lung (195)
2196	*Rousettus* sp.	F	1	Lung (123)
2199	*Rousettus* sp.	F	1	Lung (74)
2202	*Rousettus* sp.	F	1	Lung (864), liver (368), kidney (342), intestine (254), heart (807), blood (1)
9428	*Rousettus* sp.	F	2	Lung (156)
9434	*Rousettus* sp.	F	1	Lung (554), spleen (3,014), kidney (380), heart (320), intestine (2751)
9435	*Rousettus* sp.	F	2	Lung (127)
9442	*E. spelaea*	M	2	Lung (180), spleen (88)
9445	*Rousettus* sp.	M	1	Lung (143)
9447	*Rousettus* sp.	F	2	Lung (132), liver (154), spleen (661)
9447	Rousettus sp.	F	1	Lung (106,606), liver (220,051), spleen (523,582), kidney (41,653), brain (4,885), heart (17,982), intestine (11,788), blood (485)
9454	*Rousettus* sp.	M	1	Lung (448)
9457	*E. spelaea*	M	1	Lung (52)
9459	*Rousettus* sp.	F	2	Lung (182)
9463	*E. spelaea*	M	2	Lung (114)

*These represent primers and probes designed based on partial sequences of the virus L gene obtained in this study. Sequence information is provided in the online Technical Appendix (https://wwwnc.cdc.gov/EID/article/23/3/16-1119-Techapp1.pdf).

To detect filovirus IgG and IgM, we expressed His-tagged truncated nucleoproteins from RESTV or ZEBOV in *Escherichia coli* and used them as antigens (online Technical Appendix). In this experiment, we used 25 bat samples from 2015 that had enough serum volume for testing; 14 samples showed a strong cross-reaction with the ZEBOV nucleoprotein, and among them, 7 showed a weak cross-reaction with RESTV nucleoprotein. We used Western blotting to confirm these results; 11 of the 25 samples were positive for ZEBOV nucleoprotein and 4 for RESTV nucleoprotein (Table 1; Technical Appendix Figure 2). No samples overlapped between those identified as positive by PCR and those identified as positive by serologic testing. Results of a serum neutralization assay with HIV pseudovirus carrying the ZEBOV glycoprotein showed that the ELISA-positive samples had no cross-neutralization activity to the pseudovirus (*14*).

## Conclusions

We detected novel filovirus sequences with high divergence in *E. spelaea* and *Rousettus* sp. bats in China. Phylogenetic analysis of partial sequences suggested that at least 3 distinct groups of filovirus are circulating in fruit bats in China. The distances between these sequences indicates that the 3 groups may represent 3 novel species or genera. Of interest, we detected antibodies reacting more strongly to ZEBOV than RESTV nucleoprotein in some filovirus RNA–negative samples, suggesting that the bats were infected with another/other filovirus(es) cross-reactive with ZEBOV nucleoprotein or that nucleoproteins of the novel filoviruses were cross-reactive with ZEBOV and RESTV nucleoproteins.

The bat samples in this study were collected from 2 caves in 2009 and 2015, respectively; the caves are ≈200 km metric apart. Across the 2 different years and locations, we detected closely related viruses and found 1 bat that was acutely co-infected by 4 different filoviruses; this finding suggests that these viruses have been circulating in the 2 bat species and that densely populated bat caves provide opportunity for cross-infection with different viruses. However, considering the migration ability of the fruit bat, we cannot exclude the possibility that there are exchanges of virus between the bats in these two caves. Longitudinal surveillance with tracking tags may help to better understand the spatial–temporal distribution of these viruses in bat populations.

In previous reports, filoviruses were primarily detected in liver and spleen tissues (*4**,**15*). In our study, we primarily detected filoviruses in the lung. We suspect that lung tissues are the major target for these bat filoviruses. Thus, these filoviruses may have the potential to be transmitted through the respiratory tract.

These results will be helpful in providing a better understanding of the distribution and diversity of filoviruses, which may have implications for public health. Considering their feeding habitats, fruit bats are often in close contact with domestic animals and human populations. It is therefore necessary to establish long-term and proactive surveillance of these viruses and related diseases.

Technical Appendix Detailed methods and primer sequences used in a study of genetically diverse filoviruses in *Rousettus* and *Eonycteris* spp. bats, China, 2009 and 2015.
