# Supplementary material for: Genetically Diverse Filoviruses in Rousettus and Eonycteris spp. Bats, China, 2009 and 2015
Source: Emerg Infect Dis. 2017 Mar;23(3):482–6. doi: 10.3201/eid2303.161119 (PMC5382765; doi:10.3201/eid2303.161119)
Supplement: Technical Appendix — Detailed methods and primer sequences used in a study of genetically diverse filoviruses in Rousettus and Eonycteris spp. bats, China, 2009 and 2015. [file 16-1119-Techapp-s1.pdf]

Article DOI: http://dx.doi.org/10.3201/eid2303.161119

Genetically Diverse Filoviruses in *Rousettus* and *Eonycteris* spp. Bats, China, 2009 and 2015

Technical Appendix

Sample Collection

Bats were trapped by mist net in Jinghong (N21°46′17,” E101°30′22,” H882 m) and Mengla (N22°26′16,” E100°41′10,” H1300 m) in Yunnan Province, China, in November 2009 and December 2015. All bats were humanely sacrificed with isoflurane and their blood and tissue samples (hearts, intestines, spleens, lungs, kidneys, livers, and brains) were immediately collected. To minimize the influence to bat colony, juvenile bats were not included in the sampling. All the samples were transported to the laboratory and stored at –80°C until use. All sampling processes were performed by veterinarians with approval from the Animal Ethics Committee of the Wuhan Institute of Virology (Animal ethics approval number: WIVA05201202) and Yunnan Institute of Endemic Diseases Control and Prevention (Animal ethics approval number: 200801 and 201302).

RNA Extraction, Filovirus Detection, and Sequencing

Bat tissue samples (≈0.1 g) were cut into small pieces and homogenized in 1 mL of Minimum Essential Medium (MEM, GIBCO) using two steel beads and shaking on a Tissue Lyser II (Qiagen). RNA was extracted from 200 μL of supernatant of ground tissues with a High Pure Viral RNA Kit (Roche) following the manufacturer’s instructions. RNA was eluted in 50 μL elution buffer and then aliquoted and stored at –80°C. Nested RT-PCR using degenerate

primers (FV-F1/R1 and FV-F2/R2) for filovirus L gene was performed to detect filovirus sequences (*1*). A first round of PCR was performed in a 25-μL reaction mix containing 12.5 μL PCR 2× reaction mix buffer, 10 pmol of each primer, 2.5 mmol/L MgSO4, 20 U RNase inhibitor, 1 μL SuperScript III/ Platinum Taq Enzyme Mix, and 5 μL RNA. The PCR program was performed as follows: 50°C for 30 min and 94°C for 4 min; 36 cycles at 94°C for 30 sec, 52°C for 30 sec, and 68°C for 40 sec; and a final extension at 68°C for 5 min. The second round of PCR was done in a 25-μL reaction mix containing 2.5 μL PCR 10× reaction buffer, 10 pmol of each primer, 50 mmol/L $MgCl_2$, 0.5 mmol/L dNTPs, 0.1 μL Platinum Taq Enzyme (Invitrogen), and 1 μL first-round PCR product. The amplification procedure was performed as follows: 94°C for 5 min; 36 cycles at 94°C for 30 sec, 52°C for 30 sec, and 72°C for 40 sec; and a final extension at 72°C for 5 min. The amplicons were gel purified and sequenced directly using the ABI prism sequencer or cloned using pGEM-T Easy Vector System for sequencing if the direct sequencing failed (Technical Appendix Figure 1).

In vitro RNA Transcription and Establishment of Standard Curve

Based on the sequences obtained in this study, we designed 3 sets of primers and probes that specifically target the three groups of filovirus detected in China (Technical Appendix Table). Probes were labeled at the 5′ end with 6-carboxyfluorescein (6-FAM) and at the 3′ end with Black Hole Quencher 1 (BHQ1). Primers targeting fragments of templates from each of the 3 groups were synthesized (Technical Appendix Table). The forward primer contained a 5′-T7 RNA polymerase promoter sequence (TAATACGACTCACTATAGGG) to facilitate in vitro transcription. The template DNA was amplified from positive samples and transcribed into RNA with a MAXIscript Kit (Applied Biosystems), according to the manufacturer’s instructions. The RNA transcripts were diluted with a 10-fold serial dilution and used as standards to calculate viral genome copy number.

Viral RNA Quantification

We performed qRT-PCRs with AgPath-ID One-Step RT-PCR Kit (Applied Biosystems) according to the manufacturer's instructions. Each 25-μL reaction mixture contained 12.5 μL of 2× RT-PCR buffer, 1 μL RT-PCR buffer enzyme mix, 400 nmol/L each primer, 120 nmol/L probe, and 3 μL of RNA extract. Amplification was carried out in 96-well plates with Step One PCR instructions (Applied Biosystems). Thermocycling conditions were as follows: 10 min at 45°C for reverse transcription; 10 min at 95°C for activation of Taq DNA polymerase; and 40 cycles at 95°C for 15 sec, 54°C for 20 sec, and 68°C for 20 sec. Each run included 3 viral positive template controls and 2 negative controls to monitor performance. Positive samples were characterized by a well-defined exponential fluorescence curve that crossed the cycle threshold (C_t) within 36 cycles. Specimens with a C_t >36 were repeated to exclude operational faults. Viral genome copy number was calculated in each sample using the standard curves of the template RNA.

Antibody Detection by ELISA

The nucleocapsid (NP) gene of RESTV and EBOV were synthesized based on reference sequences with reference numbers FJ621583 and L11365, respectively. Predicted epitope of NP were cloned to pPET28a (Sequence will be provided upon request). The His-tagged truncated NP of RESTV (Reston-NP) or EBOV (Zaire-NP) were expressed in *Escherichia coli* BL21 and purified with a His.Bind Kit (Novagen) following the manufacturer's instructions. The purified proteins strongly reacted to hyperimmune rabbit sera raised against the full-length NP protein of RESTV or EBOV by ELISA (*2*), and no cross reactive with each other. Purified truncated NPs from EBOV or RESTV were coated on ELISA plates at ≈100 ng/well, and bat sera were tested in triplicate at a dilution of 1:100, followed by detection with horseradish peroxidase (HRP)-conjugated protein A/G (Pierce) at a dilution of 1:20,000. Samples with a mean optical density at least 3-fold higher than that of the negative control were considered positive. A positive sample

and a negative sample from our previous results were used as a positive and negative control, respectively (*2*).

Antibody Detection by Western Blotting

Truncated EBOV or RESTV NPs (500 ng) were separated through sodium dodecyl sulfate polyacrylamide gel electrophoresis (SDS-PAGE). Proteins were transferred onto a membrane made of polyvinylidene difluoride, which was activated with methanol. The membrane was then blocked with 3% bovine serum albumin in phosphate buffered saline for 1 h at 37°C. After blocking, the membrane was incubated with bat serum at a dilution of 1:100 overnight at 4°C. Rabbit serum immunized with truncated EBOV or RESTV NP was used as the positive control. The membrane was incubated with HRP-conjugated protein A/G (Pierce) at 1:40,000. The HRP substrate (Millipore) was added onto the blot and detected under chemiluminescent camera (Technical Appendix Figure 2). The positive serum samples were further confirmed by neutralization assay with pseudovirus with EBOV glycoprotein (GP) as previously reported (*2*).

References

1. He B, Feng Y, Zhang H, Xu L, Yang W, Zhang Y, et al. Filovirus RNA in fruit bats, China. Emerg Infect Dis. 2015;21:1675–7. PubMed http://dx.doi.org/10.3201/eid2109.150260

2. Yuan J, Zhang Y, Li J, Zhang Y, Wang LF, Shi Z. Serological evidence of ebolavirus infection in bats, China. Virol J. 2012;9:236. PubMed http://dx.doi.org/10.1186/1743-422X-9-236

Technical Appendix Table. Primer sequences used for template preparation and viral quantification

Primer	Sequence*
Group1-Te-F	*TAATACGACTCACTATAGGG* ATCAAGCGTCGTGGCATCG
Group1-Te-R	ACAGGAGATGCAGGTCCAAAG
Group2-Te-F	*TAATACGACTCACTATAGG* ATGCAGGTCCATAGCTTC
Group2-Te-R	TCATCAAGCGTCGTGGCAT
Group3-Te-F	*TAATACGACTCACTATAGG* GCATCAAGCGTCATGGCA
Group3-Te-R	CTCTACTCCTCCTAAGTGAC
Group1-Q-F	TGATCCCATTATGTTATATGCAT
Group1-Q-R	TCCTTCTATCCCTCCAAGATG
Group1-Q-Probe	Fam-TAGTGATTATTATAGCCCTCCTT-BHQ1
Group2-Q-F	CCCACATGATAGTAGTAACC
Group2-Q-R	TGAGTGACCTGTACTGTCCTC
Group2-Q-Probe	Fam-TTAGCACACATTGGAGGATC-BHQ1
Group3-Q-F	ACAATCCACCTCACTGTCTAA
Group3-Q-R	CTCTACTCCTCCTAAGTGAC
Group3-Q-Probe	Fam-AAGCCAAAATCGAGAACATCCA-BHQ1

*The T7 promoter sequence is italicized.

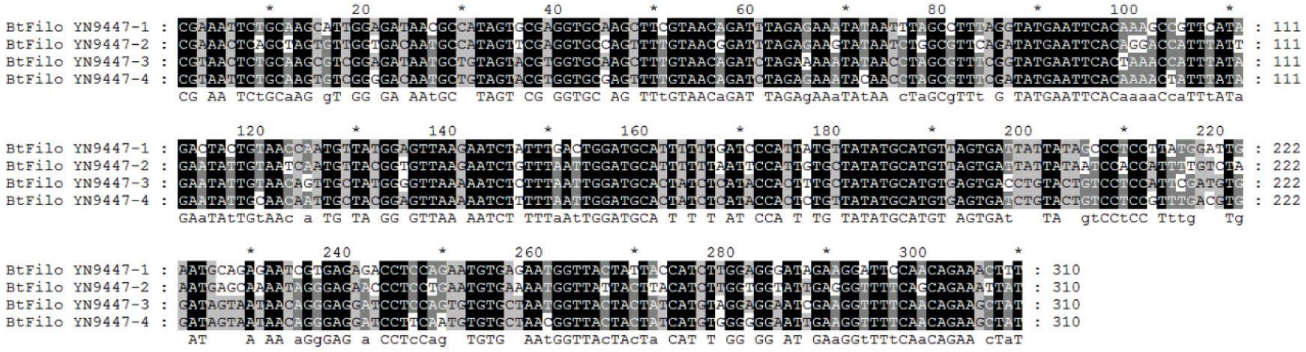

Technical Appendix Figure 1. Alignment of different strains of filovirus YN9447.

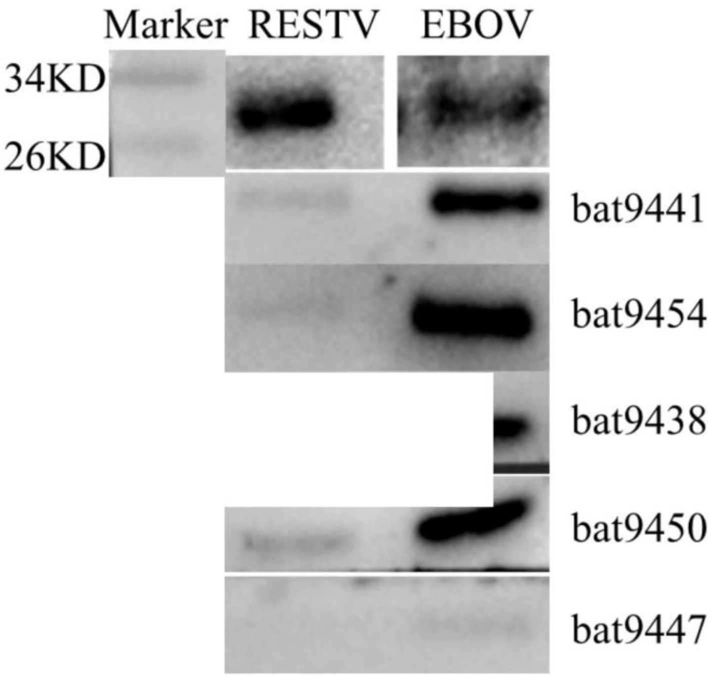

Technical Appendix figure 2. Confirmation of positive serum samples by Western blotting. Truncated EBOV- or RESTV-NPs (500 ng) were transferred onto a polyvinylidene fluoride membrane. The membrane was incubated with bat serum at a dilution of 1:100 and detected with HRP-conjugated protein A/G (Pierce) at 1:40,000. Polyclonal antibodies against the full-length NP of RESTV or EBOV were used as positive controls.
